# *Moringa oleifera* Leaf Extract Ameliorates Photooxidative Damage and Photoaging Induced by Ultraviolet-B in HaCaT Keratinocytes

**DOI:** 10.3390/antiox14070766

**Published:** 2025-06-22

**Authors:** Tanaporn Hengpratom, Benjawan Dunkhunthod, Kittipot Sirichaiwetchakoon, Pimchaya Prompradit, Issara Chaisit, Mariena Ketudat-Cairns, Salila Pengthaisong, James R. Ketudat-Cairns, Yothin Teethaisong

**Affiliations:** 1 Division of Health and Applied Sciences, Faculty of Science, Prince of Songkla University, Songkhla 90110, Thailand; tanaporn.he@psu.ac.th; 2 Thai Traditional Medicine Program, Faculty of Nursing and Allied Health Sciences, Phetchaburi Rajabhat University, Phetchaburi 76000, Thailand; benjawan.dun@mail.pbru.ac.th; 3 Division of Pharmacology and Biopharmaceutical Sciences, Faculty of Pharmaceutical Sciences, Burapha University, Chon Buri 20131, Thailand; kittipot.si@go.buu.ac.th; 4 Department of Medical Sciences, Faculty of Allied Health Sciences, Burapha University, Chon Buri 20131, Thailand; pimchy.p@gmail.com (P.P.); aungissara1909@gmail.com (I.C.); 5 School of Biotechnology, Institute of Agricultural Technology, Suranaree University of Technology, Nakhon Ratchasima 30000, Thailand; ketudat@sut.ac.th; 6 School of Chemistry, Institute of Science, Suranaree University of Technology, Nakhon Ratchasima 30000, Thailand; salila@sut.ac.th (S.P.); cairns@sut.ac.th (J.R.K.-C.)

**Keywords:** *Moringa oleifera*, UVB, photooxidative damage, photoaging, HaCaT keratinocytes

## Abstract

Skin damage and premature aging are predominantly driven by UV radiation through several mechanisms. The most common of these are by reactive oxygen species (ROS) generation, upregulation of matrix metalloproteinases (MMPs), and weakened antioxidant defenses. *Moringa oleifera* is a nutritionally valuable plant with diverse biological activities. This study optimized ethanol concentrations coupled with ultrasonic-assisted extraction to maximize the yield and efficacy of *M. oleifera* leaf extract (MOLE). We also elucidated the underlying mechanisms of MOLE in protecting against photooxidative damage and skin aging from UVB exposure in HaCaT keratinocytes. Extraction with 50% ethanol produced the highest total phenolic and flavonoid contents, aligning with the greatest antioxidant activity by ABTS and FRAP assays. MOLE showed no significant cytotoxicity up to 1000 µg/mL in the MTT assay. MOLE protected cells from detrimental UVB radiation by scavenging ROS; reducing cell damage and death; enhancing gene expression of superoxide dismutase (*SOD*-1), glutathione peroxidase (*GPx*), and catalase (*CAT*); and improving SOD activity. UVB exposure elevated *MMP*-1, *MMP*-3, and *MMP*-9 expression and decreased collagen type I (*col*-1) and elastin (*ELN*) expression, while these effects were ameliorated by MOLE. Our findings suggest that MOLE protected against UVB-induced photooxidative damage and premature aging in the HaCaT keratinocytes.

## 1. Introduction

The skin serves as the body’s first barrier against environmental damage, microorganisms, and water loss. Skin health is influenced by environmental, genetic, and lifestyle factors. Its deterioration leads to wrinkles, loss of elasticity, dryness, discoloration, and a rough texture. Skin aging is a common condition caused by intrinsic and extrinsic factors. Intrinsic aging involves natural physiological or chronological changes, leading to a thinner epidermis, reduced basal cell proliferation, and alterations in extracellular matrix (ECM) [1,2]. Extrinsic skin aging is predominantly driven by UV radiation, which accounts for 80% of facial aging. UV rays penetrate the dermis, resulting in altering the extracellular matrix (ECM), leading to thin skin and loss of connective tissue. Skin aging involves three major classes of biomolecules: proteoglycans, glycosaminoglycan (hyaluronan), and structural proteins (fibrillin, fibronectin, laminin, collagen, and elastin), all of which are affected by UV radiation [3,4].

UVB (280–320 nm) is the predominant cause of skin damage by generating reactive oxygen species (ROS) and triggering the upregulation of matrix metalloproteinases (MMPs) to degrade ECM, such as collagen and elastin [5]. Additionally, AP-1 suppresses TGF-β, reducing type I and III procollagen expression. Chronic UV exposure accelerates collagen loss, contributing to wrinkles and photoaging [6], and deteriorates the activity of endogenous antioxidant enzymes, including superoxide dismutase (SOD), catalase (CAT), and glutathione peroxidase (GPx) in epidermis and dermis up to 26–93% [7,8]. Suppression of ROS and MMP activities, together with strengthening antioxidant defense, are promising strategies to ameliorate the detrimental effects of UVB radiation.

Moringa (*Moringa oleifera* Lam.), a plant belonging to Moringaceae family, is an edible plant possessing diverse nutritional and health benefits. It is commonly found across Thailand and other tropical regions. Beyond its use as a food ingredient, distinct parts of Moringa (leaves, seeds, bark, roots, and flowers) have been utilized for alleviating inflammation, infections, hypertension, and diabetes [9,10,11]. In addition, 70% ethanol *Moringa oleifera* leaf extract (MOLE) in topical formulation has been reported to ameliorate atopic dermatitis in a BALB/c mice model [12]. A study on the antioxidant effects of Moringa leaf and pod extracts in rats found that these extracts boost the level of glutathione (GSH), a key compound in cellular defense against oxidative damage, while also reducing malondialdehyde (MDA) levels. Furthermore, ethanolic extracts from Moringa pods demonstrated strong free radical scavenging activity [13]. Previous studies have demonstrated that water-based extraction stimulates dermal fibroblast proliferation, thereby accelerating the healing process [14]. Similarly, Gothai et al. (2016) found that Moringa leaf extract obtained through ethyl acetate extraction also facilitates wound healing by promoting dermal fibroblast growth [15]. A study on Moringa stem extract in HaCaT cells showed that it has antioxidant properties, boosting antioxidant enzyme activity and activating PPARα in keratinocytes [16]. The primary bioactive compounds that contribute to skin health are phenolic compounds, including flavonoids, while the antioxidant properties are mainly attributed to vitamins A, C, and E [17].

The techniques reported to prepare Moringa extracts were mostly by organic solvent extraction [18]. Enhancing extraction efficiency and optimizing extraction conditions are important to maximize effectiveness of the extracts. Those previous studies did not employ ultrasonic-assisted extraction (UAE) and optimize the extraction conditions for efficiently extracting distinct parts of *M. oleifera*. No studies have reported the protective effects of *M. oleifera* extracted by a UAE technique, particularly *M. oleifera* leaf extract (MOLE), on UVB-induced photooxidative damage and skin aging in HaCaT human skin keratinocytes. The present study, therefore, employed a UAE technique, coupled with varying ethanol concentrations, to optimize the extraction of bioactive compounds from *M. oleifera* leaves, pods, and seeds. The extract with the highest phenolic and flavonoid content was further evaluated for its antioxidant capacity and photoprotective effects in HaCaT keratinocytes. The underlying mechanisms of action in preventing oxidative damage and skin photoaging in human skin HaCaT keratinocytes upon UVB exposure were also elucidated in the present study.

## 2. Materials and Methods

### 2.1. Plant Extraction

The fresh leaves, pod, and seeds of *Moringa oleifera* Lam. (Moringaceae) were bought from the local market, Nakhon Ratchasima Province, Thailand. A voucher specimen of the plant (T. Thummavongsa 2024-1) was deposited at Nakhon Ratchasima Rajabhat University Herbarium. Fresh leaves, pods, and seeds of *M. oleifera* were washed and dried in a hot air oven at 50 °C. The dried pieces were ground into powder. In total, 100 grams of the powder were extracted with water alone or 30%, 50%, 70%, and 100% *v*/*v* ethanol in water in an ultrasonic water bath at a frequency of 35 kHz with peak ultrasonic output of 860 watts (Sonoex DT 514 BH; Bandelin Electronic, Berlin, Germany) for 30 min. The extracts were concentrated by the rotary evaporator and lyophilized to obtain the dried *M. oleifera* extracts (MOEs). The crude extracts from the different solvents were stored at −20 °C until use.

### 2.2. Total Phenolic Content (TPC)

The TPCs of the different parts of *M. oleifera* extracts (MOEs) were analyzed by the Folin–Ciocalteu method as originally described by Singleton et al. (1999) [19]. Briefly, 20 µL of the MOE or gallic acid (standard) solutions were mixed with distilled water, 10% Folin–Ciocalteu reagent, and 7.5% sodium carbonate [19]. The mixture was left to stand at room temperature for 2 h before its absorbance was measured spectrophotometrically at 760 nm, and the results were expressed as mg gallic acid equivalent per gram of extract (mg GAE/g extract).

### 2.3. Total Flavonoid Content (TFC)

The TFCs of the different parts of *M. oleifera* extracts (MOEs) were analyzed by the aluminum chloride method [20]. Briefly, 20 µL of extract or quercetin (a standard flavonoid), 10 µL of 10% aluminum chloride (AlCl_3_), 160 µL of ethanol, and 10 µL of 1 M sodium acetate were mixed together. The mixture was incubated in the dark at room temperature for 40 min. The absorbance was measured at 415 nm. Total flavonoid content was expressed as mg quercetin equivalent per gram of extract (mg QE/g extract).

### 2.4. Antioxidant Activity of MOEs

#### 2.4.1. ABTS Radical Scavenging Assay

An ABTS assay was used to determine antioxidant capacities of leaf, seed, and pod extracts of *M. oleifera* (MOEs) [21]. An ABTS free radical was prepared by mixing 7 mM ABTS with 2.45 mM potassium persulfate (K_2_S_2_O_8_) solution in a ratio of 1:0.5 for 12–16 h in the dark. The ABTS free radical solution was diluted with ethanol to achieve an absorbance of 0.7 ± 0.05 at 734 nm. Subsequently, an aliquot of 20 µL of the MOE at various concentrations was added to 180 µL of ABTS free radical cation solution, mixed thoroughly, and incubated for 6 min at room temperature away from light. The absorbance was measured at 734 nm. The percentage of inhibition was calculated according to the following formula:

Inhibition (%) = ((A₀ − Aₛ)/A₀) × 100%

where A_0_ is the absorbance of the blank and A_s_ is the absorbance of the sample.

The IC_50_ values of MOEs were calculated from linear regression.

#### 2.4.2. Ferric Reducing Antioxidant Power (FRAP) Assay

The reducing power of MOEs was determined by a ferric reducing ability of plasma (FRAP) assay. The FRAP reagent was freshly prepared by mixing a 300 mM acetate buffer (pH 3.6), a 20 mM FeCl_3_·6H_2_O solution, and a 10 mM TPTZ solution (dissolved in 40 mM HCl) in a ratio of 10:1:1, respectively. The MOE was mixed with the FRAP reagent, shaken well, and left to stand for 30 min. The absorbance of the reaction was measured at 593 nm. Ethanolic solutions of Fe(II) (prepared using FeSO_4_) were used to create a calibration curve. The IC_50_ of each MOEs was calculated from linear regression.

### 2.5. Cell Culture

HaCaT keratinocytes, a human skin keratinocyte cell line, were purchased from CLS Cell Lines Service GmbH (Eppelheim, Germany). The cells were cultured in 5% CO_2_ at 37 °C in Dulbecco’s Modified Eagle’s Medium (DMEM) (Gibco™, Billings, MT, USA) containing 10% fetal bovine serum (FBS), 1% HEPES, and 100 U of penicillin–streptomycin antibiotics.

### 2.6. Cytotoxicity Effects of MOLE

HaCaT cells were seeded in a 96-well plate (cell culture polystyrene and flat bottom plate, Corning Life Science, Wujiang, China) at a density of 1.5 × 10^4^ cells/well and incubated at 37 °C for 24 h. Then, 0.2% DMSO (vehicle control) or MOLE at varying concentrations in 0.2% DMSO were added and further incubated at 37 °C for 24 h. Afterward, the plate was centrifuged at 1500 rpm for 5 min, and the culture medium was removed. The MTT (0.5 µg/mL) was added to each well, and the plate was incubated for an additional 4 h. The MTT reagent was then removed, and 100% DMSO was added to dissolve the formazan crystals. The absorbance was measured at 540 nm, and the percent viability was calculated in comparison to the control group, which was arbitrarily assigned 100% viability in the formula below. The MTT assay was performed in three independent experiments, with triplicate wells for each condition.

Cell viability (%) = (Absorbance of treated cells/Absorbance of control cells) × 100%

### 2.7. Photoprotective Effect of MOLE upon UVB Irradiation

To assess the effects of MOLE on protection of cell viability and damage induced by UVB-irradiation, the cell viability was determined by the MTT and cellular alteration visualized under the light microscope based on the method described in a previous study [22]. HaCaT cells were cultured in a 96-well plate at a density of 1.5 × 10^4^ cells/well and incubated at 37 °C for 24 h. The cells were pretreated with MOLE for 2 h, followed by exposure to UVB irradiation (75 mJ/cm^2^ with the plate lid removed) and incubated for 24 h. Cell viability (%) was determined and calculated as in the MTT assay. Furthermore, the cell morphological changes and damage were monitored and captured by inspection under an inverted light microscope (Nikon Eclipse Ts2, Tokyo, Japan).

### 2.8. Reactive Oxygen Species (ROS)

ROS levels were determined with the fluorescent probe, DCFH-DA, in accordance with a previous study with slight modifications [23]. Briefly, cells were seeded (1.5 × 10^4^ cells/well) in a black and clear bottom 96-well plate (Corning Life Science, Jiangsu, China) for 24 h, followed by treating the cells with MOLE (50, 100, and 200 µg/mL) and 3 mM N-acetyl cysteine (Nac) for 24 h. After incubation, cells were incubated with 50 μM H_2_DCF-DA in the Hanks’ Balanced Salt Solution (HBSS) (Gibco, USA) at 37 °C for 60 min in the dark and subsequently exposed to UVB (75 mJ/cm^2^). Cells were washed with PBS prior to measuring fluorescence intensity in a fluorescence microplate reader (EnSigh, PerkinElmer Inc., Waltham, MA, USA) with an excitation wavelength of 485 nm and an emission wavelength of 535 nm. The percentage of DCF fluorescence intensity was calculated by the following formula:

DCF fluorescence intensity (%) = (DCF fluorescence intensity test group/DCF fluorescence intensity control group) × 100%.

### 2.9. Gene Expression Analysis by Quantitative Reverse Transcription PCR (RT-qPCR)

The expression levels of genes related to skin aging, including matrix metalloproteinases (MMPs), *MMP*-1, *MMP*-3, and *MMP*-9; collagen type 1 (*col*-1); and elastin (*ELN*), as well as genes associated with the endogenous antioxidant pathway, such as superoxide dismutase-1 (*SOD*-1), glutathione peroxidase (*GPx*), and catalase (*CAT*), were analyzed by RT-qPCR. The primer set is listed in Supplementary Table S1. Briefly, HaCaT cells were cultured in a 6-well plate at a density of 4.5 × 10^5^ cell/well and incubated at 37 °C for 24 h. Then, the cells were pretreated with MOLE for 2 h, followed by UVB irradiation at 50 mJ/cm^2^. After incubation for 18 h, RNA was extracted by Trizol^TM^ reagent (Thermo Fisher Scientific, Bremen, Germany) according to manufacturer’s protocol. The contaminated genomic DNA was removed by DNase digestion. Total RNA (5 µg) was reverse-transcribed into cDNA via the Viva cDNA synthesis kit (Vivantis Technologies, Selangor, Malaysia). A total of 50 ng cDNA was used as a template in real-time PCR using the LightCycler^®^ 480 SYBR green I master mix (Roche Diagnostics, Indianapolis, IN, USA). The PCR thermocycling was set as following: initial denaturation at 95 °C for 5 min, followed by 40 cycles of 95 °C for 10 s, 60 °C for 20 s, and 72 °C for 30 s, followed by melting curve analysis over a temperature range of 65–95 °C. Relative fold-changed *mRNA* expression was calculated by the 2^−ΔΔCt^ method [24]. GAPDH was used as a housekeeping gene. The melting curve analysis of each amplicon was also done to validate specificity.

### 2.10. Superoxide Dismutase (SOD) Activity

HaCaT cells were seeded in a 6-well plate (4.5 × 10^5^ cells/well) for 24 h. The cells were pretreated with MOLE at distinct concentrations of MOLE (50, 100, and 200 µg/mL) for 2 h before exposure to UVB (50 mJ/cm^2^). After incubation for 24 h, cells were washed with PBS prior to extracting proteins in Chaps cell extraction buffer (Cell Signalling Technology, Danvers, MA, USA) supplemented with protease inhibitor cocktails (Sigma-Aldrich, Saint Louis, MO, USA). The total protein content was determined by the BCA Protein Assay (Novagen^®^, EMD Millipore Corp., Burlington, MA, USA). A total of 100 mg proteins was used to evaluate the activity of the SOD enzyme by following the manufacturer’s instructions (Dojindo Laboratories, Kumamoto, Japan).

### 2.11. Gas Chromatography-Mass Spectrophotometry (GC-MS)

The chemical constituents in *M. oleifera* leaf extract (MOLE) were evaluated by GC-MS in an Agilent 7890A GC system and Agilent 7000B MS (Agilent Technologies, Santa Clara, CA, USA), equipped with HP-5 capillary column (30 m × 0.32 mm, 0.25 um). Briefly, 2 µL of MOLE (100 mg/mL in ethanol) was injected by autosampler in 5:1 split injection mode, 250 °C for injection temperature, and 1.0 mL/min helium for column flow. The column temperature was initiated at 40 °C for 5 min, followed by 200 °C for 25 min, and 280 °C for 61 min. The MS was operated with electron ionization as an ion source and an ion source temperature of 230 °C, 70 eV electron energy, and 50–650 mass scanning mode. Data analysis and peak matching were operated using the National Institute of Standards and Technology mass spectrometry search program v.2.0 (National Institute of Standards and Technology, Gaithersburg, MD, USA) 

### 2.12. Statistical Analysis

Data from 3 replicates were presented as means ± standard deviation (SD). Statistically significant differences (*p* < 0.05) were analyzed by one-way ANOVA followed by Duncan’s post hoc test. All analyses were performed in SPSS version 23 (SPSS Inc., Chicago, IL, USA).

## 3. Results

### 3.1. Total Phenolic Content (TPC) of MOEs

**Figure 1:**
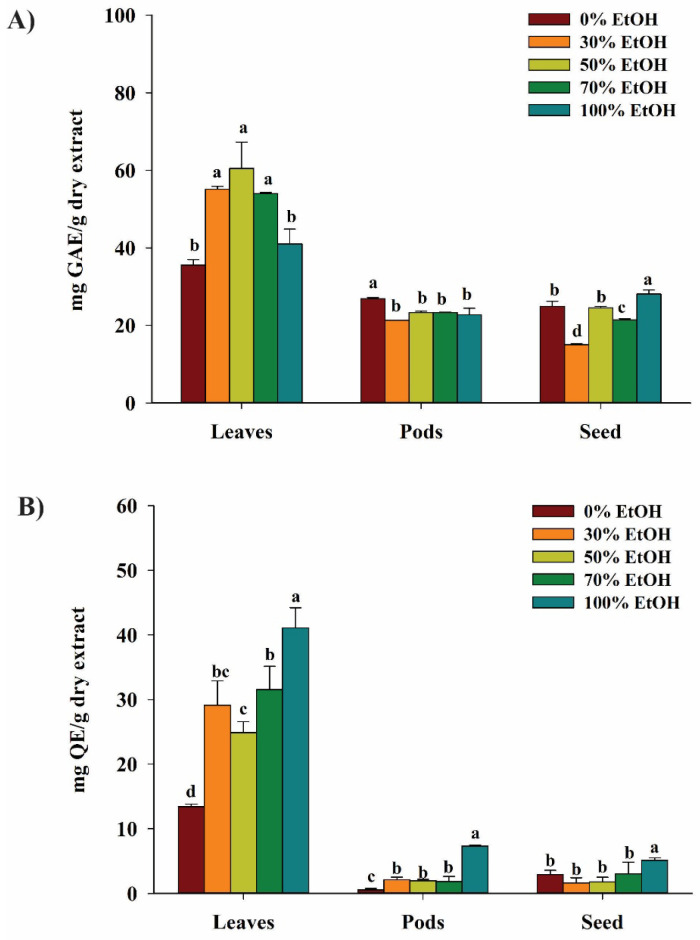
Total phenolic contents (TPCs) and total flavonoid contents (TFCs) of leaf, pod, and seed extracts of *M. oleifera* extracted using various concentrations of ethanol in distilled water (0%, 30%, 50%, 70%, and 100%). (**A**) TPCs of leaves, pods, and seed extracts are expressed as milligrams of gallic acid equivalent (GAE) per gram of extract (mg GAE/g extract). (**B**) TFCs of leaf, pod, and seed extracts are expressed as milligrams of quercetin equivalent (QE) per gram of extract (mg QE/g extract). Data are presented as means ± SD (n = 3). Different letters on the graph indicate statistically significant differences (*p* < 0.05) analyzed by one-way ANOVA followed by Duncan’s post hoc test.

The TPCs of leaves, pods, and seed extracts quantified based on the standard curve of gallic acid (Supplementary Figure S1) showed that *M. oleifera* leaf extract (MOLE) contained the highest total phenolic content (TPC) compared to pods and seeds (Figure 1A). Extraction of leaves with 50%, 30%, and 70% exhibited the highest TPC at 55.3 ± 6.1, 55.1 ± 0.8, and 54.0 ± 0.3 mg GAE/g, respectively. Aqueous extracts of pods showed a significantly higher TPC (26.9 ± 0.4 mg GAE/g) (*p* < 0.05) compared to pods extracted with ethanol, while the highest TPC of seed extract was found in 100% ethanolic extract at 28.1 ± 1.0 mg GAE/g, followed by water (24.9 ± 1.3 mg GAE/g) and 50% ethanol (24.5 ± 0.4 mg GAE/g). The results indicated that *M. oleifera* leaf extract with 50% ethanol provided the highest TPC.

### 3.2. Total Flavonoid Contents (TFCs) of MOEs

The TFCs of leaf, pod, and seed extracts of *M. oleifera* in different concentrations of ethanol were calculated from the standard curve of quercetin (QE) (Supplementary Figure S2). The leaves of *M. oleifera* contained higher TFC than pods and seeds (Figure 1B). In addition, leaves extracted with 100% ethanol significantly (*p* < 0.05) exhibited the highest total flavonoid content at 41.1 ± 3.1 mg QE/g. Similarly, the pod and seed extracts with 100% ethanol showed the highest TFC for each tissue at 7.3 ± 0.2 and 12.7 ± 2.7 mg QE/g, respectively. The results indicated that the highest TFC was found in leaves extracted with 100% of the ethanol.

### 3.3. Antioxidant Activity of MOE

In this study, the antioxidant activities of *M. oleifera* leaf extracts with 50% ethanol were strongest, as judged by ABTS (2,2′-azino-bis (3-ethylbenzothiazoline-6-sulfonic acid)) and FRAP (ferric reducing antioxidant power) assays, with an IC_50_ of 72 µg/mL from the ABTS assay and 181 µg/mL from the FRAP assay (Table 1). Likewise, 50% ethanolic pod extract showed the strongest effect in both ABTS and FRAP assays, but pod extract activity was lower than that of leaf extract. The highest free radical scavenging activity of the seed extract was found in 100% ethanol, as presented in Table 1. The findings demonstrated that the ethanolic extract of leaves, particularly extraction with 50% ethanol, coupled with UAE, possessed the highest antioxidant activity among the samples tested. Consequently, this extract was selected for further subsequent experiments.

### 3.4. Cytotoxicity Effects of M. oleifera Leaf Extract (MOLE)

**Figure 2:**
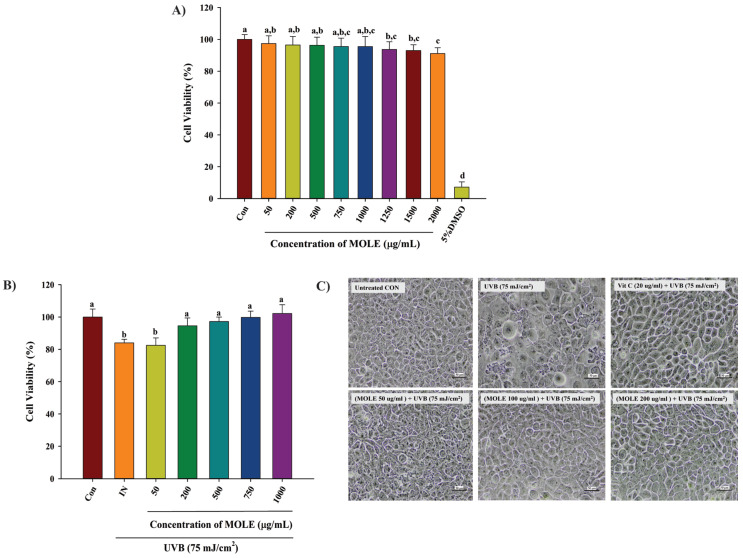
Lack of cytotoxicity of MOLE on HaCaT cells. (**A**) Cytotoxic effects of *Moringa oleifera* leaf extract (MOLE) on HaCaT cells were evaluated by the MTT assay. (**B**) The protective effects of *M. oleifera* leaf extract (MOLE) against UVB-induced damage in HaCaT keratinocytes. (**C**) Cell damage visualized under an inverted light microscope (scale bars = 50 μm, magnification 400×). Con = control (untreated cells); IN = UVB irradiation; MOLE 50 to 200 μg/mL = *M. oleifera* leaf extracts at concentrations of 50 to 200 μg/mL; 5% DMSO = 5% dimethyl sulfoxide (positive control group, induced cell death). Data are presented as means ± SD (n = 6). Different letters on the graph indicate statistically significant differences (*p* < 0.05) analyzed by the one-way ANOVA followed by Duncan’s post hoc test.

The MTT assay showed that human HaCaT keratinocyte cells treated with MOLE at concentrations up to 1000 µg/mL showed no significant (*p* < 0.05) reduction in cell viability compared to the untreated control (Con) (Figure 2A). However, exposure to concentrations exceeding 1000 µg/mL resulted in significantly increased cell death compared to the untreated control. Dramatic reduction in cell viability was seen in cells treated with 5% DMSO, a control for cell death. The results indicate that MOLE concentrations up to 1000 µg/mL were considered safe. Non-toxic concentrations were selected for subsequent experiments.

### 3.5. Protective Effects of MOLE Against UVB-Induced Cell Damage

The protective effect of MOLE upon UVB radiation evaluated by the MTT assay demonstrated that exposure to UVB at a dose of 75 mJ/cm^2^ (Induction; IN) significantly (*p* < 0.05) reduced the cell survival rate compared to untreated cells (untreated control; Con) (Figure 2B). However, pretreatment with MOLE at 200 to 1000 µg/mL significantly prevented the reduction of cell viability induced by UVB induction, while no significant difference was observed between non-irradiated control cells and cells treated with 200 to 1000 µg/mL prior to UVB irradiation. In addition, this photoprotective effect of MOLE was confirmed by observation of no substantial cell damage in cells pretreated with MOLE compared to control, while UVB-exposed cells without MOLE pretreatment exhibited cell damage and abnormal cell morphology, as depicted in Figure 2C. These findings indicate that MOLE can protect against skin damage from UVB radiation.

### 3.6. Effects of MOLE on UVB-Induced Reactive Oxygen Species (ROS)

**Figure 3:**
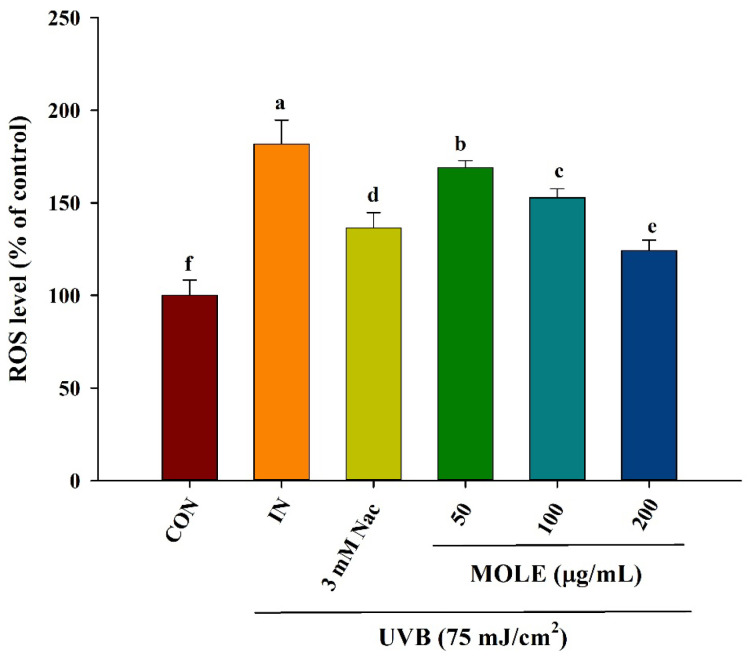
The effects of *Moringa oleifera* leaf extract (MOLE) on intracellular reactive oxygen species (ROS) in HaCat cells upon UVB exposure. The cells were pretreated with Nac (3 mM) or MOLE (50, 100, and 200 μg/mL). CON = control (untreated cells); IN = induction (cells exposed to UVB radiation without treatment); MOLE 50, 100, and 200 μg/mL = *M. oleifera* leaf extract at 50, 100, and 200 μg/mL concentrations; Nac = N-acetyl cysteine. Data are presented as means ± SD (n = 3). Different letters on the graph indicate statistically significant differences (*p* < 0.05) analyzed by one-way ANOVA followed by Duncan’s post hoc test.

Cells’ exposure to UVB induces the generation of ROS [5]. In the present study, intracellular ROS levels were assessed in the control (Con), UVB-irradiated (IN), and 3 mM of Nac (a positive control), or 50, 100, and 200 μg/mL of MOLE-pretreated groups for 24 h. The results revealed UVB-exposed cells increased the accumulation of ROS, whereas cells pretreated with Nac and MOLE exhibited decreased ROS generation in a concentration-dependent manner (Figure 3).

### 3.7. Gene Expression Analysis Using RT-qPCR

**Figure 4:**
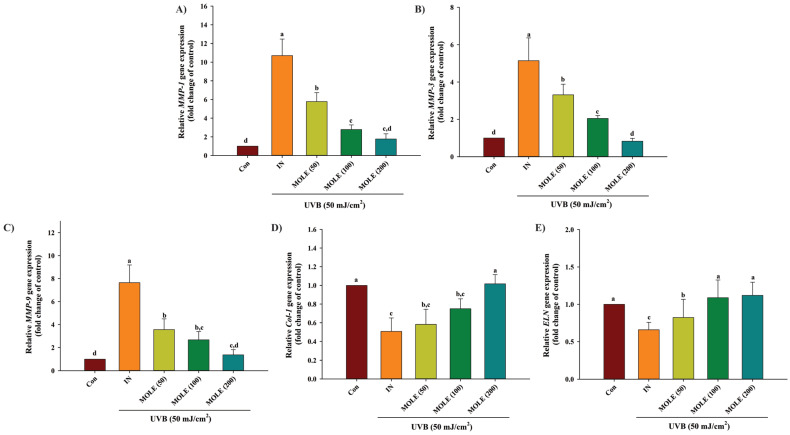
The effects of *Moringa oleifera* leaf extract (MOLE) on mRNA expression of *MMP*-1 (**A**), *MMP*-3 (**B**), *MMP*-9 (**C**), *col*-1 (**D**), and *ELN* (**E**) in HaCaT cells upon UVB irradiation. CON = control (untreated cells); IN = irradiation (cells exposed to UVB radiation without treatment); MOLE 50, 100, and 200 μg/mL = *M. oleifera* leaf extract at concentration 50, 100, and 200 μg/mL. Data presented as means ± SD (n = 3). Different letters on the graph indicate statistically significant differences (*p* < 0.05) analyzed by one-way ANOVA followed by Duncan’s post hoc test.

The *mRNA* expression analysis of the *MMP*-1, *MMP*-3, *MMP*-9, *col*-1, and *ELN* genes showed a significant increase in the expression of *MMP*-1, *MMP*-3, and *MMP*-9 upon UVB irradiation compared to the control group (non-irradiated cells) (Figure 4). However, cells pretreated with MOLE at concentrations of 50, 100, and 200 µg/mL showed significantly decreased expression of the *MMP*-1, *MMP*-3, and *MMP*-9 genes (*p* < 0.05) induced by UVB, compared to cells exposed to UVB (Figure 4A–C). The expression of the *col*-1 *and ELN* genes was significantly reduced in cells exposed to UVB, while these effects were significantly mitigated by MOLE treatments in a dose-dependent manner (Figure 4D,E). The results indicate that MOLE suppressed UVB-induced gene expression of *MMP*-1, *MMP*-3, and *MMP*-9, while the extract increased the expression of the *col*-1 and *ELN* genes in cells exposed to UVB.

**Figure 5:**
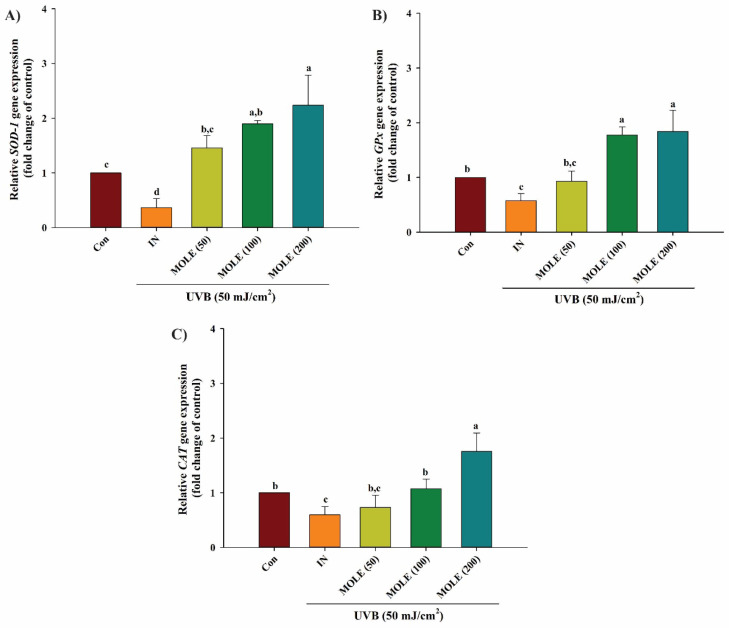
The effects of *Moringa oleifera* extract (MOLE) on the gene expression of superoxide dismutase-1 (*SOD*-1) (**A**), glutathione peroxidase (*GPx*) (**B**), and catalase (*CAT*) (**C**) in HaCaT keratinocyte human skin cells after UVB exposure. CON = control (untreated cells); IN = irradiation (cells exposed to UVB radiation without treatment); MOE 50, 100, and 200 μg/mL = *M. oleifera* leaf extract at concentrations of 50, 100, and 200 μg/mL Data are presented as means ± SD (n = 3). Different letters on the graph indicate statistically significant differences (*p* < 0.05) analyzed by one-way ANOVA followed by Duncan’s post hoc test.

The analysis of the mRNA expression of endogenous antioxidant genes, including superoxide dismustase-1 (*SOD*-1), glutathione peroxidase (*GPx*), and catalase (*CAT*), revealed that exposure to UVB radiation significantly reduced the expression of the *SOD*-1, *GPx*, and *CAT* genes when compared to the non-irradiated control group (Figure 5). However, pretreatment of cells with MOLE at concentrations of 50, 100, and 200 µg/mL significantly increased (*p* < 0.05) the expression of *SOD*-1, *GPx*, and *CAT* in a dose-dependent manner compared to the cells exposed to UVB radiation (IN) without pretreatment (Figure 5A–C).

### 3.8. Superoxide Dismutase (SOD) Activity

**Figure 6:**
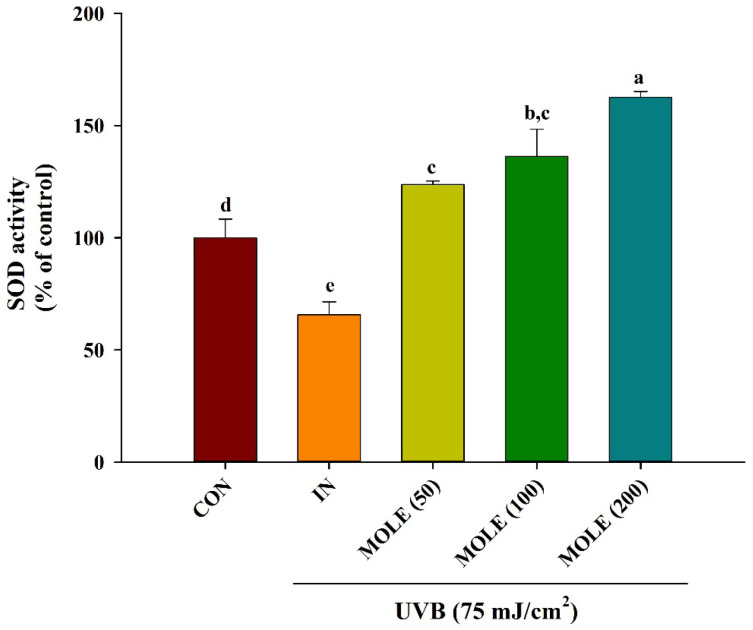
The effects of *Moringa oleifera* extract (MOLE) on superoxide dismutase (SOD) activity following UVB exposure. CON = control (untreated cells); IN = cells exposed to UVB radiation without treatment; MOLE 50, 100, and 200 μg/mL = *M. oleifera* extract at concentrations of 50, 100, and 200 μg/mL. Data are presented as means ± SD (n = 3). Different letters on the graph indicate statistically significant differences (*p* < 0.05) analyzed by one-way ANOVA followed by Duncan’s post hoc test.

To confirm the effect of MOLE on endogenous antioxidant enzymes UVB-irradiated HaCaT cells at the protein level, superoxide dismutase (SOD) activity was evaluated. The SOD activity upon UVB exposure and MOLE treatment in HaCaT cells is illustrated in Figure 6. Cells exposed to the UVB (IN) significantly reduced SOD activity compared to control untreated cells (*p* < 0.05). Pretreatments with MOLE at concentrations of 50, 100, and 200 µg/mL not only significantly prevented the reduction of SOD activity from UVB, but the extract also enhanced SOD activity in a dose-dependent manner (*p* < 0.05).

### 3.9. The GC-MS of MOLE

The chemical constituents in the MOLE were analyzed by GC-MS. The GC-MS analysis tentatively identified a total of 37 known compounds present in the MOLE, as detailed in Table 2 and Supplementary Figure S3. Among these, dodecyl acrylate (16.63%), linolenic acid (8.31%), propanoic acid 3,3′-thiobis-, didodecyl ester (7.97%), propanoic acid, 3-mercapto-dodecyl ester (7.33%), 1-dodecanol (5.46%), heptacosane (5.42%), nonacosane (4.77%), octacosane (4.48%), triacontane (3.94%), hexacosane (3.55%), *n*-hexadecanoic acid (3.51%), and pentacosane (3.41%) were the most abundant compounds that were identified in MOLE. Besides, MOLE also contained other compounds known to have remarkable biological activities, such as phytol and α-tocopherol.

## 4. Discussion

UV radiation can cause premature skin aging through multiple mechanisms, such as DNA damage, the production of reactive oxygen species (ROS), and skin inflammation [25]. Prolonged UV exposure stimulates skin cells to produce various types of free radicals, including superoxide ions, hydroxyl radicals, and hydrogen peroxide, causing oxidative stress and damage to the cells [26]. Additionally, UV radiation induces upregulation of the activity of matrix metalloproteinases (MMPs), enzymes that degrade collagen and elastin, leading to skin wrinkling [27]. It also weakens intracellular antioxidant defenses, including SOD, GPx, and CAT. The present study investigated the efficacy of MOEs, particularly MOLE, in preventing and inhibiting oxidative damage and the expression of matrix metalloproteinases, as well as their mitigating effects on endogenous antioxidant enzymes affected by detrimental UVB radiation.

This study demonstrated that extracts among various parts of the edible Moringa. MOLE contained the highest levels of total phenolic content (TPC) and total flavonoid content (TFC) compared to extracts of pods and seeds. Moreover, MOLE exhibited the highest antioxidant and free radical scavenging activity when compared to extracts from other parts. High antioxidant potential of leaf extract is aligned with its high TPC and TFC. Our findings are consistent with previous studies that Moringa possesses antioxidant properties in extracts of its leaves, seeds, and pods. Ethanol-extracted MOLE showed antioxidant activity, and phenolic, flavonoid, carotenoid, and lycopene compounds are the main active compounds found in the leaf extract [28]. Furthermore, it was found that water-extracted MOE (leaves) exhibited antioxidant activity against free radicals such as superoxide, nitric oxide, and lipid peroxidation, which is comparable to the standard antioxidant compounds [29,30].

Ultrasonic-assistant extraction (UAE) has emerged as a highly efficient extraction technique. It has been extensively used to enhance extraction yield of bioactive compounds from natural products, such as phenolic compounds, flavonoids, carotenoids, alkaloids, and terpenoids [31]. Compared to other extraction methods, UAE is less time-consuming, has low solvent requirement and high extraction yield, and is suitable for heat-labile bioactive compounds [32]. Several studies reported that UAE improves the yield of plant phytocompounds, particularly polyphenols [33,34,35]. It is important to highlight that UAE has a superior efficiency in extraction of bound phenolic compounds compared to conventional solvent extraction [36]. Here in the present study, we utilized the UAE technique with ethanolic extraction method to enhance the yield of phytochemicals, including total phenolics and total flavonoids.

The study of genes related to endogenous antioxidant enzymes, including *SOD*, *GPx*, and *CAT*, revealed that MOLE had protective and ameliorative effects on the expressions of these genes from deleterious UVB exposure. Noteworthy, the potential of MOLE on endogenous antioxidant defenses was confirmed by the enhanced SOD activity in HaCaT cells upon UVB exposure. These results are consistent with previous studies that found Moringa extract possesses protective effects on keratinocyte skin cells by enhancing the activity of various endogenous antioxidant enzymes, such as SOD and catalase [16]. Matrix metalloproteinases (MMPs) are calcium-dependent, zinc-containing enzymes that play a key role in tissue remodeling and the degradation of the ECM, targeting components such as collagen, elastin, gelatin, glycoproteins, and proteoglycans. This study found that cells exposed to UVB radiation increased the expression of the *MMP*-1, *MMP*-3, and *MMP*-9 genes while downregulating the expression of the collagen type I and elastin genes, which correspond with a previous study [37]. However, MOLE decreased expression of the *MMP*-1, *MMP*-3, and *MMP*-9 genes after UVB exposure. It was also found that MOLE effectively enhances the expression of genes that are responsible for collagen type I and elastin production.

A toxicity study of Moringa leaves revealed that the plant contains less than 12 g/kg of tannins and 21 g/kg of phytic acid (based on dry weight), with no detectable toxic compounds such as trypsin inhibitors, lectins, amylase inhibitors, or glucosinolates [38]. Small amounts of tannins were found in the pods and stems. Additionally, Moringa leaf extract has been used as a contraceptive, but the root bark should be avoided during pregnancy due to its potential to cause miscarriage [39]. According to a study by Asare et al. (2012) [40], water-extracted Moringa leaves at a concentration of 20 mg/kg showed toxicity to peripheral blood mononuclear cells. Awodele et al. (2012) conducted an acute toxicity study in rats, revealing that rats could tolerate Moringa aqueous leaf extract up to concentration of 6.4 g/kg when ingested and up to concentration of 1.5 g/kg when administered intraperitoneally [41]. In chronic toxicity tests in mice, an oral dose of 1585 mg/kg of MOLE resulted in 50% mortality (LD50), but there were no adverse effects on blood parameters or sperm when administered orally.

## 5. Conclusions

**Figure 7:**
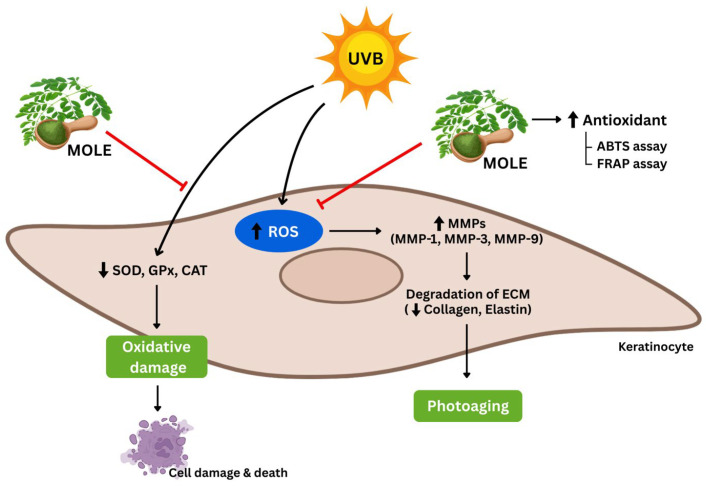
Schematic representation of proposed mechanism of action of *Moringa oleifera* leaf extract (MOLE) in ameliorating UVB-induced oxidative damage and skin photoaging in HaCaT keratinocytes.

This study demonstrated that *M. oleifera* extracts, especially those of leaf extracted in 50% ethanol coupled with UAE, show strong antioxidant capability corresponding to their total phenolic and flavonoid contents. MOLE scavenged ROS induced by UVB in HaCaT keratinocytes, as well as enhanced the expression of endogenous antioxidant enzyme genes (*SOD*, *GPx*, and *CAT*) and SOD activity in the HaCaT cells, supporting the use of MOLE in protecting oxidative stress and damage. Regarding anti-photoaging potential, MOLE suppressed the expression of matrix metalloproteinases (*MMP*-1, *MMP*-3, and *MMP*-9) and prevented reduced expression of genes for collagen type 1 and elastin upon deleterious UVB exposure in the HaCaT cells. The proposed mechanism of action of MOLE is illustrated in Figure 7. Taken together, MOLE possesses high antioxidant activity, low cytotoxicity to human skin HaCaT keratinocytes, and photoprotective effects against oxidative damage and skin aging.

## Figures and Tables

**Table 1:** Antioxidant capacities of leaf, pod, and seed extracts of *Moringa oleifera* in various ethanol concentrations, evaluated by ABTS and FRAP assays.

*Moringa oleifera* Extract (MOE)	Leaves		Pods		Seeds	
	ABTS (IC_50_, µg/mL)	FRAP (IC_50_, µg/mL)	ABTS (IC_50_, µg/mL)	FRAP (IC_50_, µg/mL)	ABTS (IC50, µg/mL)	FRAP (IC_50_, µg/mL)
0% Ethanol	121	518	257	677	379	794
30% Ethanol	74.2	300	237	603	409	986
50% Ethanol	72.1	181	224	581	387	807
70% Ethanol	88.7	286	259	689	354	784
100% Ethanol	120	227	277	724	339	731

**Table 2:** Compounds tentatively identified by GC-MS of *Moringa oleifera* leaf extract (MOLE).

No.	Compounds	RT	% Area
1	Propane, 1,1-dimethoxy	5.9	3.10
2	2-Cyclopenten-1-one, 2-hydroxy-	8.5	0.19
3	2,4-Dihydroxy-2,5-dimethyl-3(2H)-furan-3-one	9.7	0.10
4	Pyranone	13.2	0.35
5	2-Methoxy-4-vinylphenol	16.3	0.25
6	1-Dodecanol	18.9	5.46
7	Phenol, 2,4-bis(1,1-dimethylethyl)-	19.5	0.22
8	Dihydroactinidiolide	19.7	0.19
9	Dodecyl acrylate	22.0	16.63
10	4-((1E)-3-Hydroxy-1-propenyl)-2-methoxyphenol	22.8	0.32
11	Tetradecanoic acid	23.0	0.24
12	Phytol, acetate	24.0	0.13
13	2-Pentadecanone, 6,10,14-trimethyl-	24.0	0.08
14	n-Hexadecanoic acid	25.6	3.51
15	Hexadecanoic acid, ethyl ester	25.9	0.27
16	Propanoic acid, 3-mercapto-, dodecyl ester	26.2	7.33
17	Heneicosane	27.3	0.25
18	Phytol	27.5	1.97
19	Linolenic acid	27.9	8.31
20	Linolenic acid, ethyl ester	28.2	2.71
21	Docosane	28.6	0.86
22	Tricosane	30.0	2.35
23	Tetracosane	31.4	2.57
24	Pentacosane	32.9	3.41
25	Glycerol β-palmitate	33.0	1.32
26	Hexacosane	34.2	3.55
27	Linolenic acid, 2-hydroxy-1-(hydroxymethyl)ethyl ester (Z,Z,Z)-	35.6	3.36
28	Heptacosane	35.6	5.42
29	Octacosane	37.0	4.48
20	Nonacosane	38.3	4.77
31	Triacontane	39.6	3.94
32	Hentriacontane	40.9	2.33
33	α-Tocopherol	41.2	0.21
34	Dotriacontane	42.2	1.44
35	Propanoic acid, 3,3′-thiobis-, didodecyl ester	51.1	7.97

## Data Availability

The authors confirm that the data supporting the findings of this study are available within the article and its Supplementary Materials.

## Supplementary Materials

The following supporting information can be downloaded at: https://www.mdpi.com/article/10.3390/antiox14070766/s1. Table S1: Primer sets for RT-qPCR. Figure S1: Standard curve for determination of the total phenolic content. Figure S2: Standard curve for determination of the total flavonoid content. Figure S3: Gas chromatography-mass spectrometry (GC-MS) chromatograms of MOLE. The GC conditions are shown as follows: Instrument: Agilent 7890A GC with HP-5 column (30 m × 0.32 mm, 0.25 µm). Injection: 2 µL, 5:1 split mode, injector temperature 250 °C. Carrier gas: Helium, 1.0 mL/min flow rate. Oven program: Start at 40 °C for 5 min, ramp to 200 °C for 25 min, and ramp to 280 °C for 61 min.

## Abbreviations

The following abbreviations are used in this manuscript:

ABTS	2,2′-azino-bis(3-ethylbenzothiazoline-6-sulfonic acid)
CAT	Catalase
col-1	Collagen type I
ELN	Elastin
FRAP	Ferric reducing antioxidant power assay
GC-MS	Gas chromatography-mass spectrometry
GPx	Glutathione peroxidase
MDA	Malondialdehyde
MMPs	Matrix metalloproteinases
MOE	Moringa oleifera extract
MOLE	Moringa oleifera leaf extract
ROS	Reactive oxygen species
SOD	Superoxide dismutase
TFC	Total flavonoid content
TPC	Total phenolic content
UAE	Ultrasonic-assisted extraction
