# Supplementary material for: Moringa oleifera Leaf Extract Ameliorates Photooxidative Damage and Photoaging Induced by Ultraviolet-B in HaCaT Keratinocytes
Source: Antioxidants (Basel). 2025 Jun 22;14(7):766. doi: 10.3390/antiox14070766 (PMC12291669; doi:10.3390/antiox14070766)

Moringa oleifera leaf extract ameliorates photooxidative damage and photoaging induced by ultraviolet-B in HaCaT Keratinocytes

Tanaporn Hengpratom[1], Benjawan Dunkhunthod[2], Kittipot Sirichaiwetchakoon[3], Pimchaya Prompradit[6], Issara Chaisit[6], Mariena Ketudat-Cairn[4], Salila Pengthaisong[5], James R. Ketudat-Cairns[5], and Yothin Teethaisong[6*]

Supplementary Materials

Table S1: Primer sets for RT-qPCR

Target gene	Sequences (5′ to 3′)	Accession Number
MMP-1	Forward: CTACGATTCGGGGAGAAGTG	NM_002421.4
	Reverse: TCCTTGGGGTATCCGTGTAG	
MMP-3	Forward: CCTCAGGAAGCTTGAACCTG	NM_002422.5
	Reverse: GAAACCTAGGGTGTGGATG	
MMP-9	Forward: TGTACCGCTATGGTTACACTCG	NM_004994.3
	Reverse: GGCAGGGACAGTTGCTTCT	
SOD	Forward: GTGGAGAACCCAAAGGGGAGTT	NM_001322817.2
	Reverse: GTGGAGAACCCAAAGGGGAGTT	
GPx	Forward: TGTAACCAGTTCGGGAAGCA	NM_001367832.1
	Reverse: CCACTTGATGGCATTTCCC	
CAT	Forward: GTTACTCAGGTGCGGGCATTCTAT	NM_001752.4
	Reverse: GAAGTTCTTGACCGCTTTCTTCTG	
Col-1	Forward: TGACGAGACCAAGAACTG	NM_000088.4
	Reverse: CCATCCAAACCACTGAAACC	
ELN	Forward: GCCCCTGGATAAAAGACTCC	NM_001278913.2
	Reverse: GTCCTCCTGCTCCTGCTGT	
GAPDH	Forward: GTCTCCTCTGACTTCAACAGCG	NM_001357943.2
	Reverse: ACCACCCTGTTGCTGTAGCCAA	

Figure S1: Standard curve for determination of the total phenolic content

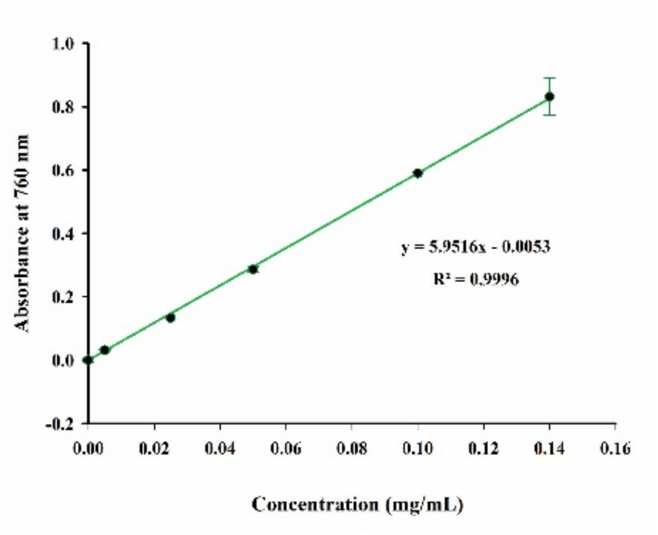

Figure S2: Standard curve for determination of the total flavonoids content

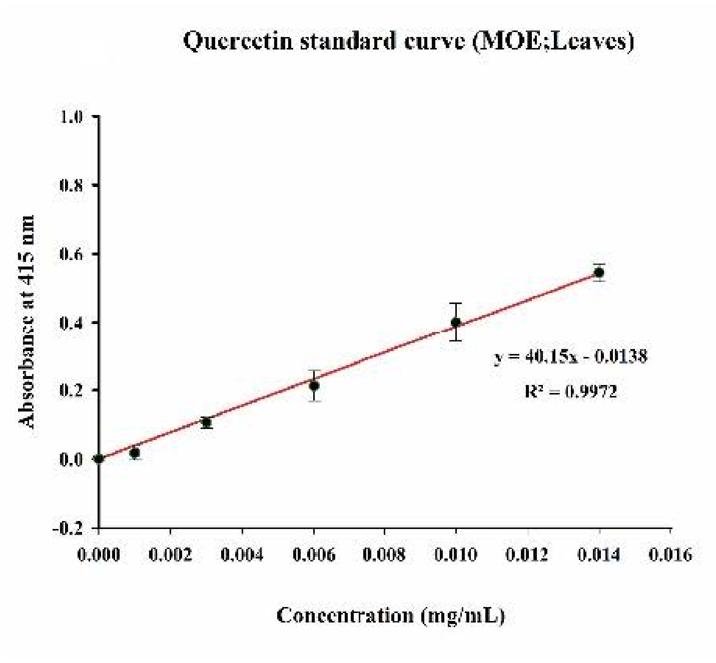

Figure S3: Gas chromatography/mass spectrometry (GC/MS) chromatograms of MOLE. The GC conditions are shown as follows: Instrument: Agilent 7890A GC with HP-5 column (30 m × 0.32 mm, 0.25 μm). Injection: 2 μL, 5:1 split mode, injector temperature 250 °C. Carrier gas: Helium, 1.0 mL/min flow rate. Oven program: Start at 40 °C for 5 min, Ramp to 200 °C for 25 min, and Ramp to 280 °C for 61 min.

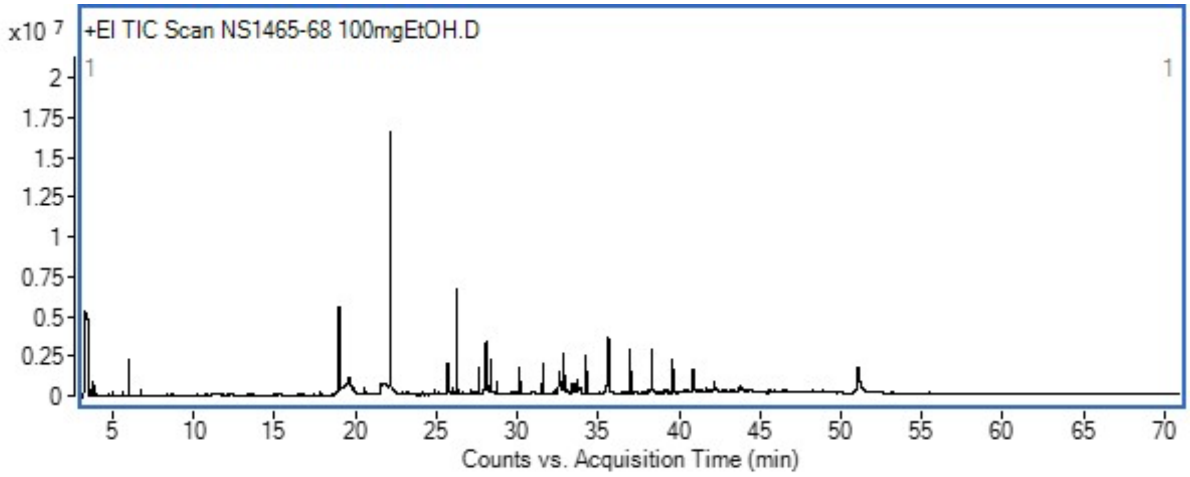

Supplement: Supplementary file 1 [file antioxidants-14-00766-s001.zip › antioxidants-3650264-supplementary.pdf]
